# Proportion, Type, and Characteristics of Physician Entrepreneurship in Massachusetts

**DOI:** 10.1001/jamanetworkopen.2020.26938

**Published:** 2021-01-06

**Authors:** Wesley H. Greenblatt

**Affiliations:** 1 Massachusetts Institute of Technology Sloan School of Management, Cambridge, Massachusetts; 2 Boston Children’s Hospital, Boston, Massachusetts

## Abstract

**Importance:**

Although there have been signs of increasing interest in entrepreneurship among physicians as well as claims of a paucity of entrepreneurial activity in health care in general, there is little systematic evidence of the extent, type, and characteristics of entrepreneurship by physicians. Physician involvement in entrepreneurship may result in more innovative and financially successful health care companies.

**Objective:**

To evaluate the proportion and characteristics of physicians who founded new businesses and the types of businesses that they started.

**Design, Setting, and Participants:**

This cross-sectional study was conducted by matching all 33 770 physicians holding a Massachusetts medical license in 2017 with the Massachusetts new business registration records from 1960 to 2017 to identify companies founded by physicians. Data were analyzed from September 2017 to December 2019.

**Main Outcomes and Measures:**

The number of physician-founded companies in Massachusetts and the types of businesses as characterized by the stated purpose at the time of founding.

**Results:**

Among the 33 770 physicians holding a Massachusetts license in 2017, 13 839 (41.0%) were women and 8029 (23.8%) were international medical graduates; the median year of graduation from medical school was 1994 (interquartile range, 1983-2004). A total of 6494 (19.2%) physicians had founded at least 1 new business, and 831 of the 2448 physicians (33.9%) who graduated from medical school between 1974 and 1978 had founded a business. A total of 9501 companies were founded, of which 6267 (66.0%) were clinical practice, real estate, or practice management companies; 703 companies (7.4%) were in the public interest, including advocacy, public health, and philanthropy; 533 (5.6%) were biotechnology, health care information technology, or medical device companies; and 1759 (18.5%) were other business pursuits. For physician entrepreneurs, the mean (SD) time from medical school graduation to company founding was 20.2 (9.8) years. Regression analysis suggested that female physicians founded companies at lower rates than male physicians (odds ratio [OR], 0.529; 95% CI, 0.494-0.567) and that there was an association between attending a top-10 medical school by National Institutes of Health research funding and starting a clinical practice (OR, 0.687; 95% CI, 0.616-0.766) or biotechnology company (OR, 4.326; 95% CI, 2.951-6.344).

**Conclusions and Relevance:**

The findings of this cross-sectional study suggest that physicians may be substantially involved in entrepreneurship, although there may be disparities by sex. Facilitation of physician entrepreneurship by policy makers, educators, and institutions may enhance medical innovation and public health.

## Key Points

**Question:**

What are the proportions and characteristics of physicians who found new businesses, and what types of businesses do they found?

**Findings:**

This cross-sectional study matched 33 770 physicians who had a Massachusetts medical license in 2017 with records of Massachusetts companies founded from 1960 to 2017 and found that 19.2% of the physicians, including 33.9% of those who graduated from medical school between 1974 and 1978, founded a business. Clinical practices were the most common business type, but many companies were focused on innovation and public health.

**Meaning:**

The findings suggest that physicians in Massachusetts are substantially involved in entrepreneurship, which is an important mechanism through which they can contribute to medical innovation and public health.

## Teaser

This cross-sectional study uses Massachusetts medical licensure and business registration records to examine the proportion of physician-founded companies and the types of businesses started.

## Introduction

In 2018, the US spent $3.6 trillion on health care, representing 17.7% of the economy.^1^ Despite its size and macroeconomic significance, US health care is characterized by large productive and allocative inefficiencies with ample evidence that increased competition and improved management practices, such as may be facilitated by entrepreneurial entry, may enhance performance.^2,3,4,5^ The health care sector is also an important source of scientific and technological innovation. The US spent an estimated $117 billion on medical research in 2012.^6^ Successful commercialization, such as may occur through a startup, is often required for an innovative idea to ultimately impact patient care.^7^ Understanding the dynamics of health care entrepreneurship has large potential welfare implications.

Some scholars have indicated there has been a relative paucity of innovation-driven entrepreneurship in health care.^8,9^ Although some health care startups may serve their local communities, such as small private practices or retail optical shops, the scholars were particularly concerned about the relative absence of startups using technological, business model, or process innovations to serve the broader health care market.^10^ Noting the persistent inefficiencies in the health care system, they have raised the following question: Why have more startups not entered to innovate on these inefficiencies and improve the overall performance of the sector? In particular, they note organizational and institutional barriers, such as difficulty measuring quality and reimbursement systems rewarding quantity, that make it difficult for health care startups to appropriate the value of their innovations.

However, there are also signs of increasing interest in health care entrepreneurship.^11^ After the founding in 2005 of the first accelerator, which offered mentoring, educational programs, and seed funding to early-stage startups in exchange for equity, there has been a rapid increase in the number of health care accelerators, with 87 separate programs in the US in 2014.^12^ These include accelerators run by hospitals and health systems, such as the Cedars-Sinai Accelerator, Boston Children’s Hospital Innovation and Digital Health Accelerator, and Texas Medical Center Accelerator, all founded within the past decade.^13^ There has also been an increased number of health care hackathons and pitch competitions. For instance, MIT Hacking Medicine and Stanford University’s health++ held their first health care hackathons in 2012 and 2016, respectively.^14,15^

Physician participation in health care startups may be substantively important in improving their innovation and financial performance. Firms founded by “user innovators” (ie, those motivated by solving their own needs or use cases, such as a cardiologist developing a new catheter), are more likely to receive venture funding and generate higher revenues.^16^ In line with this, when a federal investigation^17^ alleged that established orthopedic device firms violated the anti-kickback statute in their relationship with orthopedists, the resulting settlement mandated increased frictions to working with physician innovators. This led to a decrease in the firms’ rate of innovation and a shift away from inventions in which physician knowledge is crucial. Physicians may also bring the institutional knowledge to help navigate the regulatory process and speed product uptake.^18^

Despite the potential importance of physician entrepreneurship, there has been little evidence to inform discussion: prior studies included surveys of financiers,^19^ focus groups and interviews,^20,21^ or highly selected populations.^8,18,22^ To my knowledge, the present study is the first systematic characterization of physician entrepreneurship. Data on all licensed physicians in Massachusetts as of 2017 were matched with Massachusetts business registration records from 1960 to 2017 to identify all new businesses founded by physicians. Although the entrepreneurial activity of Massachusetts physicians is likely not representative, Massachusetts is a particularly informative setting because it is among the locations most likely to have substantial rates of innovation-driven health care startups in addition to startups serving their local communities. The aim of this study was to evaluate the proportion and characteristics of physician entrepreneurs in Massachusetts and examine the types of businesses that they started.

## Methods

### Study Population and Covariates

This cross-sectional study used 2 public administrative databases. The first was medical licensure records from the Massachusetts Board of Registration in Medicine. These records include medical school, graduation year, Massachusetts licensure year, sex, business address, and specialty. Sex was self-reported to the Board of Registration in Medicine. Limited, temporary, and inactive license holders were excluded. Doctors of osteopathic medicine were also excluded owing to potential differences from medical doctors (MDs), including their geographic location within Massachusetts. The final sample of 33 770 represents all MDs able to practice medicine in Massachusetts in 2017. The study population included physicians who retired, moved to a different state, or left clinical medicine only if they retained their Massachusetts licensure. Similarly, physicians who lived in other states and founded a company in Massachusetts but did not hold a Massachusetts medical license as of 2017 were not included. Because the study used exclusively public data, it did not meet the definition of human participant research and did not require institutional review board approval. This study followed the Strengthening the Reporting of Observational Studies in Epidemiology (STROBE) reporting guideline.

Prior studies have identified that connections to a community, such as those formed when growing up in a particular area, are important in entrepreneurship.^23^ To proxy for this, a variable for having attended a Massachusetts medical school was included, given the unique concentration of medical schools and medical entrepreneurialism in the Boston area. This study controlled for the amount of National Institutes of Health (NIH) funding received by a medical school and its teaching hospitals in 2017 using data from the NIH Research Portfolio Online Reporting Tools. Because this measure is not available for international medical graduates, it was also parameterized by a variable for a top-10 medical school by NIH funding.

### Measurement of Entrepreneurship

A second publicly available administrative database was used to measure entrepreneurship: business registration records.^24,25^ These records are created when a business registers with the Massachusetts Secretary of the Commonwealth as a corporation, limited liability company, or partnership, triggering the legal creation of the firm. Sole proprietorships are excluded from this process. Advantages of registration include tax benefits, limited liability, and credibility with customers. Because registration requires an annual fee, it signals some degree of seriousness of intent by the founder. In the present study, a founder was defined as an officer of the firm at the time of registration. Business records were queried between 1960 and 2017. Potential matches were manually verified by the author using a third piece of independent information from publicly available resources such as LinkedIn, physician biographies, company websites, and truepeoplesearch.com. In cases in which this was not possible, great care was taken to avoid false-positive matches, especially with companies not directly related to medicine, even if at the expense of more false-negative matches. To measure entrepreneurship by physicians, 31 physician-company pairs founded before medical school graduation were excluded from the analysis.

Business registration documents ask founders about their company’s purpose, providing evidence at the time of founding of anticipated business activities. These documents were used to categorize startups into the following types: biotechnology, clinical practice, community and arts, consulting, health care information technology (IT), medical business, medical devices, nonmedical business, physician groups, professional societies, public interest, and real estate and holding (further details are given in eTable 1 and eFigure 1 in the Supplement). The business-type categories were determined by me after review of 400 physician-founded companies. In addition to company purpose, the business registration records also provide year of registration, year of revocation of registration, nonprofit status, and legal jurisdiction. In particular, I noted companies with a Delaware legal jurisdiction, because Delaware is often seen as having favorable corporate laws, and this jurisdiction can be a sign of growth aspirations at the time of founding.^25^

### Statistical Analysis

In addition to descriptive statistics, multivariable regression analysis was used to help assess the associations between entrepreneurship and sex, specialty, graduation year, and medical school attended. Because the main dependent variable was a binary measure of having ever founded a company, a logit model was estimated in the primary analysis. Estimates are presented as adjusted odds ratios (ORs). Robust SEs were used throughout. Statistical significance was set at *P* < .05, and all hypothesis tests were 2-tailed. Data were analyzed from September 2017 to December 2019. All analyses were conducted using Stata statistical software, version 15.1 (StataCorp LLC).

## Results

### Study Population

Of 33 770 physicians who held a Massachusetts medical license in 2017, 13 839 (41.0%) were women, 8029 (23.8%) were international medical graduates (eTables 2 and 3 in the Supplement), 7254 (21.5%) had attended 1 of the 4 Massachusetts medical schools, and 27 265 (80.7%) had a current address in Massachusetts. The median year of graduation from medical school was 1994 (interquartile range [IQR], 1983-2004), with a median time of 5 years (IQR, 3-8 years) from graduation to Massachusetts licensure (eFigure 2 in the Supplement).

### Companies Founded by Physicians

Table 1 summarizes the characteristics of physician-founded companies. A total of 9501 Massachusetts companies were founded between 1960 and 2017 by physicians holding a Massachusetts medical license in 2017. Most of these were involved with patient care or associated supporting activities, including 4153 clinical practices directly caring for patients (43.7%), 442 provider services (4.7%), and 1672 real estate and holding companies (17.6%). Provider services report a business purpose related to practice management, billing, or contracting with insurance companies, whereas real estate and holding companies report leasing or ownership of real estate, ambulatory surgery or imaging centers, or large capital equipment such as magnetic resonance imaging machines. Although some provider service and real estate companies represent separate business endeavors, such as selling billing services to other practices or renting apartments, many appear to support a physician’s own practice.

**Table 1.** Characteristics of Physician-Founded Companies

Characteristics and types	Companies (N = 9501)^a^
Company characteristic	
Nonprofit	1077 (11.3)
Jurisdiction in Massachusetts	8952 (94.2)
Jurisdiction in Delaware	391 (4.1)
Company extant in 2017	5414 (57.0)
Time in business as of 2017, median (IQR), y	7 (3-14)
Year founded, median (IQR)	2005 (1997-2013)
Founded before Massachusetts licensure	172 (1.8)
Multiple MD founders	1192 (12.5)
MD founders, mean (10th-90th percentile), No.	1.19 (1-2)
Any female MD founder	2097 (22.1)
Any international MD founder	2639 (27.8)
Company type	
Biotechnology	155 (1.6)
Clinical practice	4153 (43.7)
Community and arts	128 (1.3)
Consulting	419 (4.4)
Health care IT	209 (2.2)
Medical business	785 (8.3)
Medical devices	169 (1.8)
Nonmedical business	555 (5.8)
Other	24 (0.3)
Physician group	80 (0.8)
Professional society	135 (1.4)
Provider services	442 (4.7)
Public interest	575 (6.1)
Real estate and holding	1672 (17.6)

Abbreviations: IQR, interquartile range; IT, information technology; MD, medical doctor.

^a^ Data are presented as number (percentage) of companies unless otherwise indicated.

Overall, 533 startups founded by physicians (5.6%) were types closely associated with innovation-driven businesses, including 155 biotechnology companies (1.6%), 209 health care IT companies (2.2%), and 169 medical device companies (1.8%). Physicians also founded 575 public interest startups related to advocacy, public health, and philanthropy (6.1%). Other business pursuits were the basis for 1759 companies (18.5%), including 419 consulting companies (4.4%), 785 companies closely related to medical business (8.3%), and 555 nonmedical companies (5.8%).

Most companies had a Massachusetts legal jurisdiction, but 391 (4.1%) filed in Delaware. There were 1077 nonprofit companies (11.3%) mainly associated with public interest, professional society, community and arts, and provider service firms involved in contracting with insurance companies.

### Rate of Entrepreneurship

In the cross-section, 6494 (19.2%) of the physicians had ever founded a company, with a similar distribution among company types (Table 2). For example, 451 physicians (1.3%) in the cross-section had founded a biotechnology, health care IT, or medical device company and 646 (1.9%) had founded a public interest company. Conditional on physicians having founded at least 1 company, the mean number of firms founded was 1.73 (10th-90th percentile, 1-3 firms) (eFigure 3 in the Supplement). Whereas 4119 physician-entrepreneurs (63.4%) founded a single company, the most prodigious physician founded 21 companies across 9 different business types.

**Table 2.** Entrepreneurship Outcomes Among Physicians With a Massachusetts Medical License in 2017

Business type	Ever founded a company, No. (%) (N = 33 770)	Startups, mean (10th-90th percentile), No.^**a**^	Time from graduation to founding of first company, mean (SD), y^**a**^
Any	6494 (19.2)	1.73 (1-3)	17.15 (9.01)
Biotechnology	136 (0.4)	1.29 (1-2)	23.86 (11.47)
Clinical practice	3860 (11.4)	1.27 (1-2)	16.19 (8.34)
Community and arts	127 (0.4)	1.04 (1-1)	22.73 (12.46)
Consulting	416 (1.2)	1.08 (1-1)	20.20 (9.73)
Medical devices	145 (0.4)	1.23 (1-2)	20.90 (10.58)
Health care IT	194 (0.6)	1.18 (1-2)	17.64 (9.48)
Medical business	697 (2.1)	1.30 (1-2)	20.12 (9.56)
Nonmedical business	480 (1.4)	1.20 (1-2)	19.93 (10.00)
Physician group	111 (0.3)	1.05 (1-1)	24.32 (8.44)
Professional society	198 (0.6)	1.05 (1-1)	21.66 (9.77)
Provider services	564 (1.7)	1.18 (1-2)	21.27 (8.38)
Public interest	646 (1.9)	1.08 (1-1)	22.66 (11.07)
Real estate and holding	1398 (4.1)	1.43 (1-2)	21.34 (9.44)

Abbreviation: IT, information technology.

^a^ Conditional on having founded at least 1 company of the given type.

**Figure 1.**
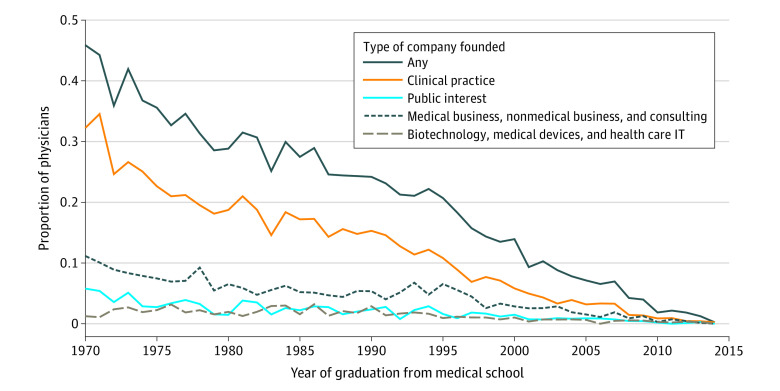
Proportion of Physicians With a Massachusetts Medical License in 2017 Who Had Ever Founded a Company, by Year of Medical School Graduation Data shown are among 33 770 physicians. IT indicates information technology.

Figure 1 summarizes the rate of entrepreneurship by medical school graduation year. For physicians nearing retirement, the probability of having ever founded a company was estimated by using the year of graduation from medical school as a proxy for age. For instance, a physician who graduated in 1978 at age 26 years would be 65 years of age in 2017. With use of this approach, 831 of the 2448 physicians (33.9%) who graduated from medical school between 1974 and 1978 (which corresponds to an age of 65 to 69 years in 2017 if they had graduated at 26 years of age) had founded at least 1 business. For this to be a valid measure, it assumes there was no association between entrepreneurship and entry into or exit from holding a Massachusetts medical license. No statistically significant difference was found in the rate of founding a company within 15 years of graduation by medical school graduation year (eFigures 4 and 5 in the Supplement).

### Age at Entrepreneurship

**Figure 2.**
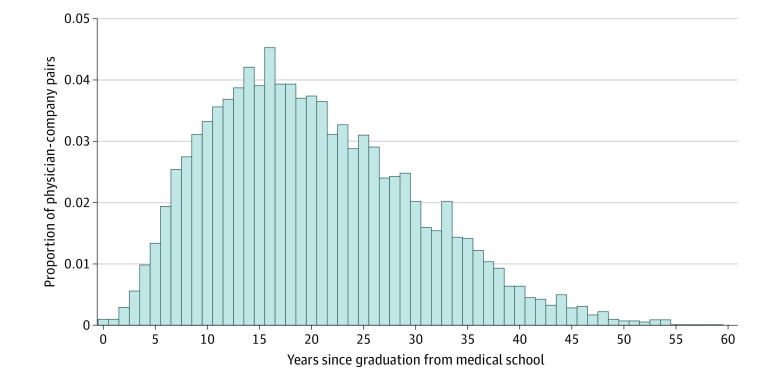
Distribution of Physician Entrepreneurship by Years Since Medical School Graduation Companies with multiple physician founders are included once for each founder.

The mean (SD) time from medical school graduation until founding a company was 20.2 (9.8) years, with a right-skewed distribution (Figure 2). If a physician graduated from medical school at age 26 years, this would put the estimated mean age at entrepreneurship at 46 years. A similar approach looks at the time between graduation and the first founding event of a given business type (Table 2). This measure would be associated with lower mean times because it excludes the age of any subsequent founding events. Clinical practices had the shortest time from graduation to founding at a mean (SD) of 16.2 (8.3) years. Physician groups and biotechnology companies had the longest mean (SD) time at 24.3 (8.4) and 23.9 (11.5) years, respectively.

### Distribution of Entrepreneurship by Physician Characteristics

**Figure 3.**
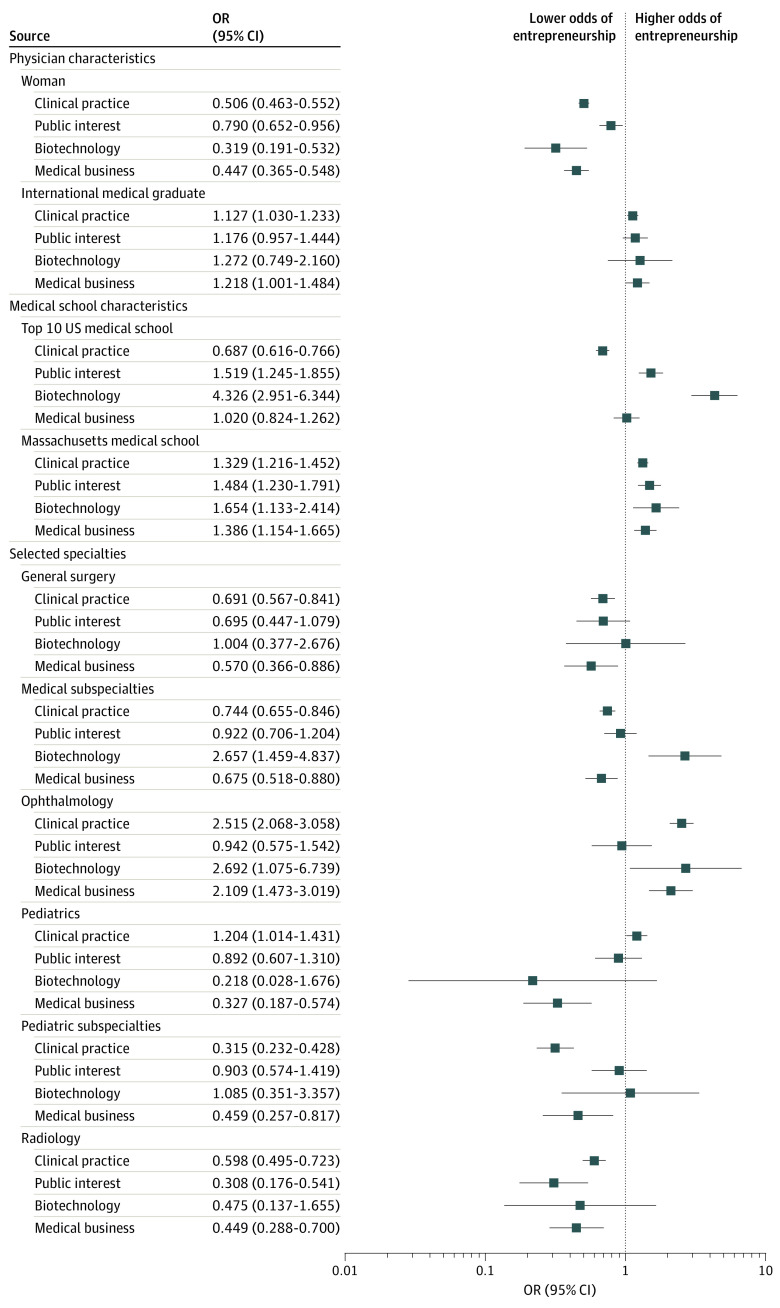
Odds of Entrepreneurship by Physician and Medical School Characteristics Select results from a logistic regression with a dependent variable of having ever founded a company of the given business type are shown. All models included 16 variables for medical specialties as well as variables for medical school graduation year categorized in 5-year periods. Specialty coefficients should be interpreted in comparison with internal medicine; 95% CIs are based on robust SEs. Squares indicate odds ratios (ORs), and horizontal lines indicate 95% CIs (full regression results are given in eTable 4 in the Supplement).

Figure 3 shows select results from a multivariable logistic regression model to understand the association between physician characteristics and the rate and type of entrepreneurship (eTables 4 and 5 in the Supplement). Overall, female physicians had a lower rate of entrepreneurship than male physicians, with an adjusted OR of 0.529 (95% CI, 0.494-0.567; *P* < .001). Female physicians had a lower rate of entrepreneurship for all business types, with the greatest disparity for businesses more closely associated with innovation (eFigure 6 in the Supplement). For instance, the adjusted OR was 0.207 (95% CI, 0.130-0.329; *P* < .001) for health care IT and 0.319 (95% CI, 0.191 - 0.532; *P* < .001) for biotechnology firms after controlling for specialty, graduation year, and medical school characteristics.

International medical graduates were more entrepreneurial than US medical school graduates (overall adjusted OR, 1.090; 95% CI, 1.012-1.175; *P* = .02). Whereas the overall figure was associated with a greater rate of clinical practice entrepreneurship, it also was associated with increased odds of founding both medical (OR, 1.218; 95% CI, 1.001-1.484; *P* = .049) and nonmedical (OR, 1.778; 95% CI, 1.429-2.214; *P* < .001) businesses.

Medical school characteristics were also associated with the rate and type of entrepreneurship. Attendees of a top-10 medical school by NIH research funding had a lower probability of founding a clinical practice (OR, 0.687; 95% CI, 0.616-0.766; *P* < .001) but a higher probability of founding a public interest (OR, 1.519; 95% CI, 1.245-1.855; *P* < .001) or biotechnology (OR, 4.326; 95% CI, 2.951-6.344; *P* < .001) firm.

There were different patterns of entrepreneurship by specialty, measured in comparison with general internal medicine physicians. For instance, among neurosurgeons, the OR of founding a medical device company compared with internists was 6.971 (95% CI, 2.316-20.938; *P* < .001); among psychiatrists, the OR was 0.288 (95% CI, 0.062-1.332; *P* = .11). Other patterns of entrepreneurship were more complex; for example, pediatricians, general surgeons, and radiologists had lower rates of founding companies across multiple business types compared with internists. For smaller specialties and rarer entrepreneurial outcomes, some results were imprecisely estimated.

## Discussion

This study found that physicians in Massachusetts were substantially involved in entrepreneurship, with 19.2% of all licensed physicians in 2017 and 33.9% of those graduating from medical school between 1974 and 1978 having registered at least 1 new company. This included founding clinical practices, which can entail substantial risk and requires strong managerial skills, as well as innovation-driven entrepreneurship. It may be helpful to contextualize the magnitude of this number by looking at entrepreneurship rates in other populations. Alumni surveys suggest an approximately 33% lifetime probability of entrepreneurship for Massachusetts Institute of Technology alumni and a cross-sectional probability of approximately 25% for Stanford Graduate School of Business alumni.^26,27^ Care must be taken in directly comparing these results given the differences in methods and types of companies founded.

This study’s findings regarding age and disparities mirror those found in other settings. If medical school graduation is assumed to occur at age 26 years, the estimated mean age among all physician entrepreneurs would be 46 years, with an estimated mean age of 42 years among clinical practice entrepreneurs and 50 years among biotechnology company entrepreneurs. By comparison, in the general population, the mean (SD) age of founders of companies that hire at least 1 employee is 42 (12.0) years, and startups in the top 0.1% by growth have a mean (SD) founder age of 45 (10.7) years.^28^ Similarly, across the economy, women are approximately half as likely as men to start a business, and immigrants have overall higher rates of entrepreneurship.^29,30,31^

Given the high failure rate among startups, a strength of using business registration data is that it allows for systematic measurement of startups that otherwise might not appear in sources based on payroll or financing events, and it avoids the biases of surveys or self-reporting. The use of business registration records also allows for rich microdata. For instance, whereas Massachusetts physicians founded 4153 clinical practices, they also founded 49 companies providing support to clinical trials, 13 marijuana-related companies, 3 breweries, and 1 mango import business. A corresponding weakness of using business registration data, however, was that some of the companies were legal entities created primarily for tax, liability, or other organizational reasons rather than representing true entrepreneurial activity. For instance, some provider service firms were legal entities to support a physician’s own private-practice billing activities, whereas others were entrepreneurial endeavors seeking to sell billing services to other practices. Similarly, some companies may represent a new legal registration of an existing organization. As an example, a clinical practice might choose to create a new registration if it transitions from a professional corporation to a limited liability company. The degree to which this occurs varies by business type.

Clinical care and entrepreneurship are not necessarily dichotomous career paths; much of the value physicians bring to potential entrepreneurial endeavors stems directly from their clinical expertise and involvement in patient care. The flexibility and relative job security of many clinical positions may help mitigate the risks of entrepreneurship. At the same time, physicians have avenues other than entrepreneurship through which they can contribute to bringing innovative ideas to market, including licensing or employment with established firms, publications, conferences, and consulting. There is little evidence as to what extent these activities may be seen as substitutes in facilitating health care innovation.

A number of potential policy implications emerged from this study. In light of previous studies showing that entrepreneurial and management training improves outcomes, the frequency of entrepreneurship suggests that there might be a greater role for more such educational programs for physicians.^32^ It remains an open question what the optimal timing of such programs may be, including during medical school, during graduate medical education, or on an as-needed basis once in practice. To the extent that policy makers may want to encourage physician entrepreneurship, policy or institutional changes may help support this. For example, prior literature has shown that credit constraints limit entrepreneurship.^33^ It is likely that medical school debt is associated with decreased entrepreneurship. Similarly, the results on the rate and type of entrepreneurship by specialty raise questions about to what extent these differences are associated with market size, expertise, culture, selection, salary, job flexibility, and other factors. Employers or professional societies may be able to stimulate entrepreneurship by developing processes and cultures that support and value entrepreneurial contributions.^34^ In some cases, these new companies will present conflicts of interest; it will be important for future research to understand the extent and implications of such conflicts and how they intersect with existing disclosure requirements and prohibitions. The sex disparities found in this study will require deliberate policies and interventions.

### Limitations

This study has limitations. First is the cross-sectional nature of the data and the potential for bias if entrepreneurship was associated with acquiring or relinquishing a Massachusetts medical license. However, no statistically significant association between medical school graduation year and the frequency of entrepreneurship within 15 years of graduation was revealed. Second, this study was not able to distinguish the degree of founder effort. The startups might represent a full-time professional activity, a side job, or a hobby. A few individuals listed on registration documents were likely included owing to a familial tie with the true founder, but this number appeared to be small. An important avenue for future research includes examining the success of physician-founded firms and their broader impact on medicine and their communities.

The results, especially those pertaining to innovation-driven businesses, may not fully generalize to other settings. Few states have business registration data allowing founder identification, limiting extension of this approach to other geographies. Massachusetts, however, both has a strong health care industry and is a leading state for venture capital funding, making it an ideal location to study physician entrepreneurship.^35^

## Conclusions

The findings of this cross-sectional study suggest that physicians in Massachusetts may be substantially involved in entrepreneurship, which may be an important mechanism through which physicians advance patient care, develop and disseminate innovation, and contribute to the economy and their communities. Much work remains, however, to understand the antecedents and consequences of physician entrepreneurship.

## Notes

Supplement. **eFigure 1.** Example excerpt from Articles of Organization **eFigure 2.** Physician count by year of graduation and medical licensure **eFigure 3.** Distribution of startup count per founder **eFigure 4.** Time trend for entrepreneurship within 15 years of graduation **eFigure 5.** Kaplan-Meier curves by year of graduation **eFigure 6.** Gender disparity by type of entrepreneurship **eTable 1.** Overview of business types **eTable 2.** Physician characteristics **eTable 3.** Physician characteristics by founding status **eTable 4.** Entrepreneurship outcomes: Logistic regression **eTable 5.** Alternative logistic model specifications
