# Supplementary material for: Proportion, Type, and Characteristics of Physician Entrepreneurship in Massachusetts
Source: JAMA Netw Open. 2021 Jan 6;4(1):e2026938. doi: 10.1001/jamanetworkopen.2020.26938 (PMC7788471; doi:10.1001/jamanetworkopen.2020.26938)
Supplement: Supplement. — eFigure 1. Example excerpt from Articles of Organization eFigure 2. Physician count by year of graduation and medical licensure eFigure 3. Distribution of startup count per founder eFigure 4. Time trend for entrepreneurship within 15 years of graduation eFigure 5. Kaplan-Meier curves by year of graduation eFigure 6. Gender disparity by type of entrepreneurship eTable 1. Overview of business types eTable 2. Physician characteristics eTable 3. Physician characteristics by founding status eTable 4. Entrepreneurship outcomes: Logistic regression eTable 5. Alternative logistic model specifications [file jamanetwopen-e2026938-s001.pdf]

Supplementary Online Content

Greenblatt WH. Proportion, type, and characteristics of physician entrepreneurship in Massachusetts. *JAMA Netw Open*. 2021;4(1):e2026938. doi:10.1001/jamanetworkopen.2020.26938

eFigure 1. Example excerpt from Articles of Organization

eFigure 2. Physician count by year of graduation and medical licensure

eFigure 3. Distribution of startup count per founder

eFigure 4. Time trend for entrepreneurship within 15 years of graduation

eFigure 5. Kaplan-Meier curves by year of graduation

eFigure 6. Gender disparity by type of entrepreneurship

eTable 1. Overview of business types

eTable 2. Physician characteristics

eTable 3. Physician characteristics by founding status

eTable 4. Entrepreneurship outcomes: Logistic regression

eTable 5. Alternative logistic model specifications

This supplementary material has been provided by the authors to give readers additional information about their work.

© 2021 Greenblatt. *JAMA Network Open*.

eFigure 1: Example excerpt from Articles of Organization

The Commonwealth of Massachusetts

William Francis Galvin

Secretary of the Commonwealth

One Ashburton Place, Boston, Massachusetts 02108-1512

ARTICLES OF ORGANIZATION

(General Laws, Chapter 156B)

ARTICLE I

The exact name of the corporation is:

Cardionomics, Inc.

ARTICLE II

The purpose of the corporation is to engage in the following business activities:

(1) To develop and market a variety of products and services for the treatment of cardiovascular disease utilizing advances in gene delivery, gene therapy, micro-array technologies and other target identification and screening technologies; and
(2) To carry on any business permitted by the laws of the Commonwealth of Massachusetts to a corporation organized under CHapter 156B of the Massachusetts General Laws.

Note: Excerpt from a publicly available business registration document demonstrating the purpose of the business.

© 2021 Greenblatt. *JAMA Network Open*.

eFigure 2: Physician count by year of graduation and medical licensure

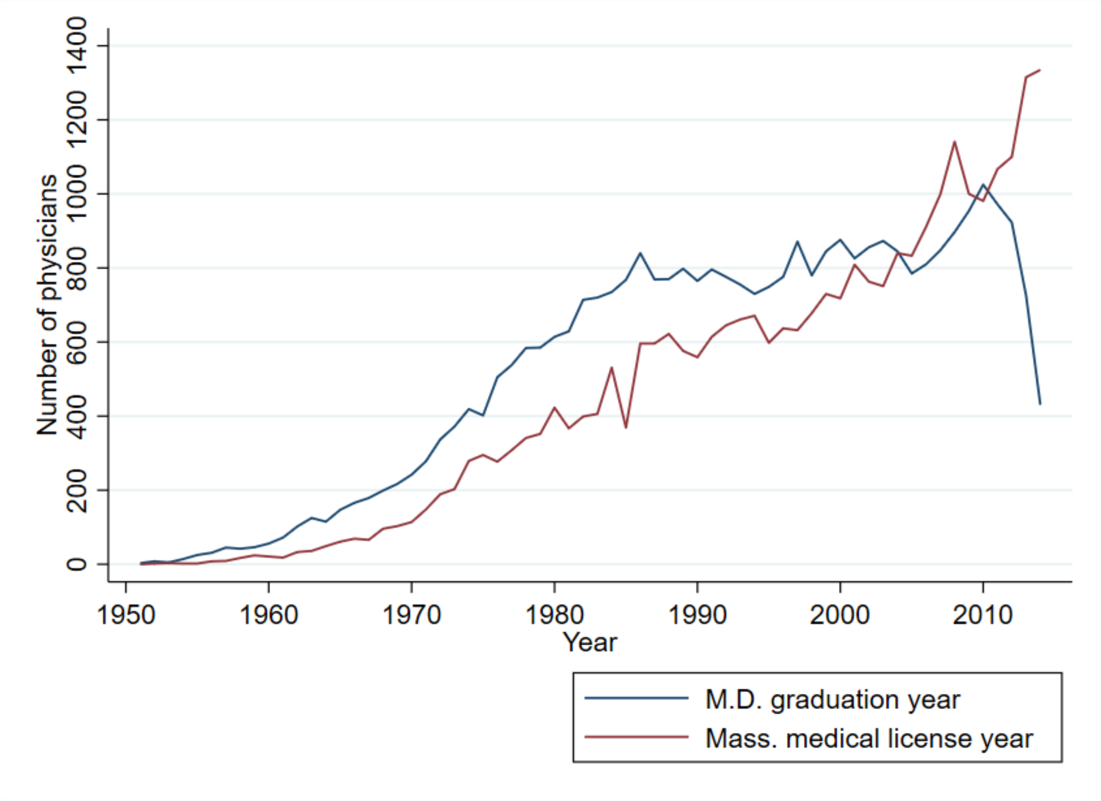

<u>Note</u>: N = 33,770 physicians holding a Massachusetts medical license in 2017.

© 2021 Greenblatt. *JAMA Network Open*.

eFigure 3: Distribution of startup count per founder

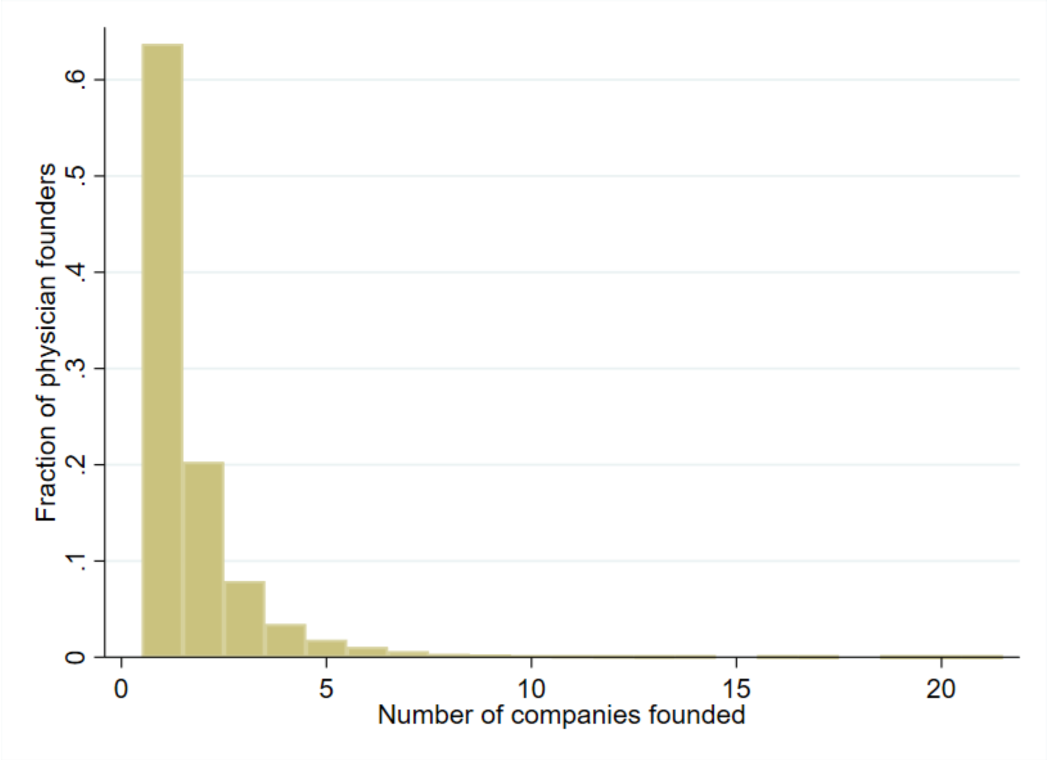

Note: Distribution limited to physicians founding at least one company.

© 2021 Greenblatt. *JAMA Network Open*.

eFigure 4: Time trend for entrepreneurship within 15 years of graduation

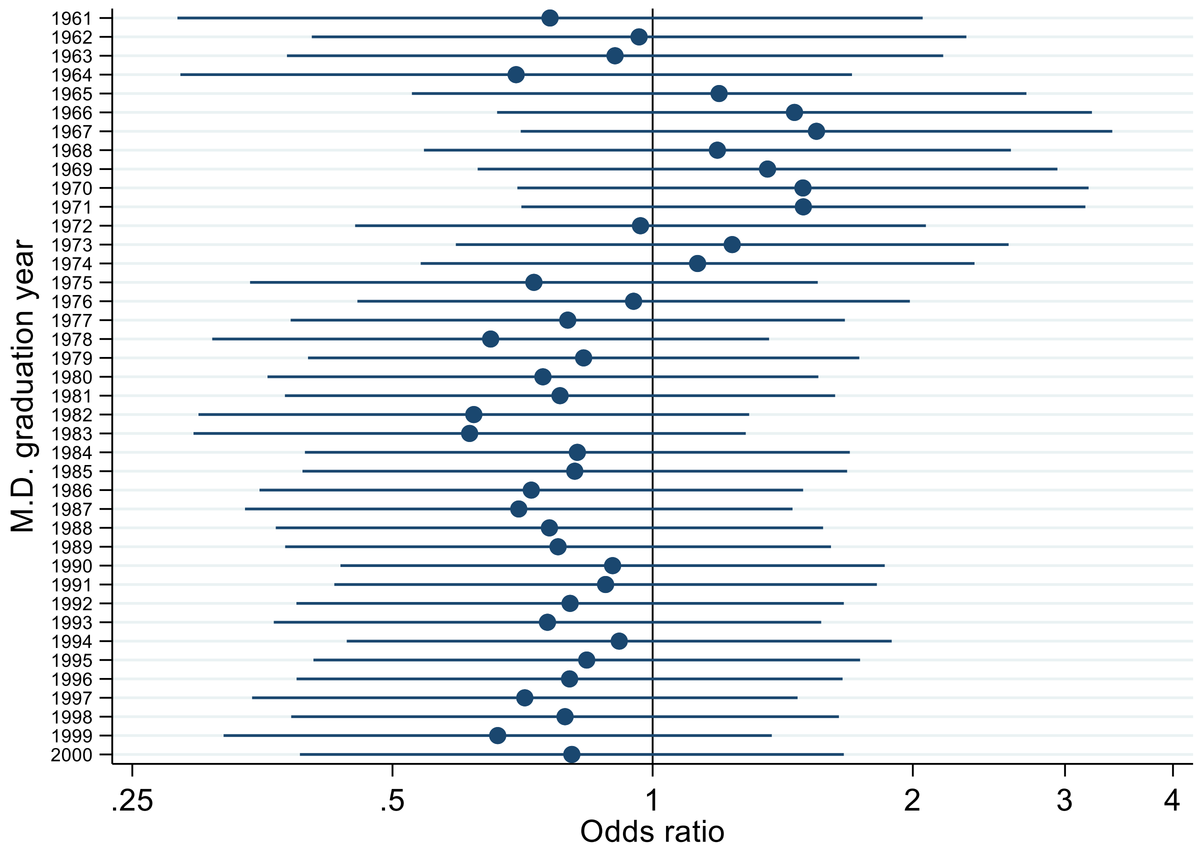

Note: The coefficients on indicator variables for year of graduation from a logistic regression are plotted above. The dependent variable is founding any company within 15 years of medical school graduation. The odds ratio is reported relative to graduating in 1960. The regression also includes indicator variables for gender, international medical graduate, Massachusetts medical school, top 10 medical school, and a set of 21 medical specialties. Robust standard errors are used. Error bars denote the 95% confidence interval.

© 2021 Greenblatt. *JAMA Network Open*.

eFigure 5: Kaplan-Meier curves by year of graduation

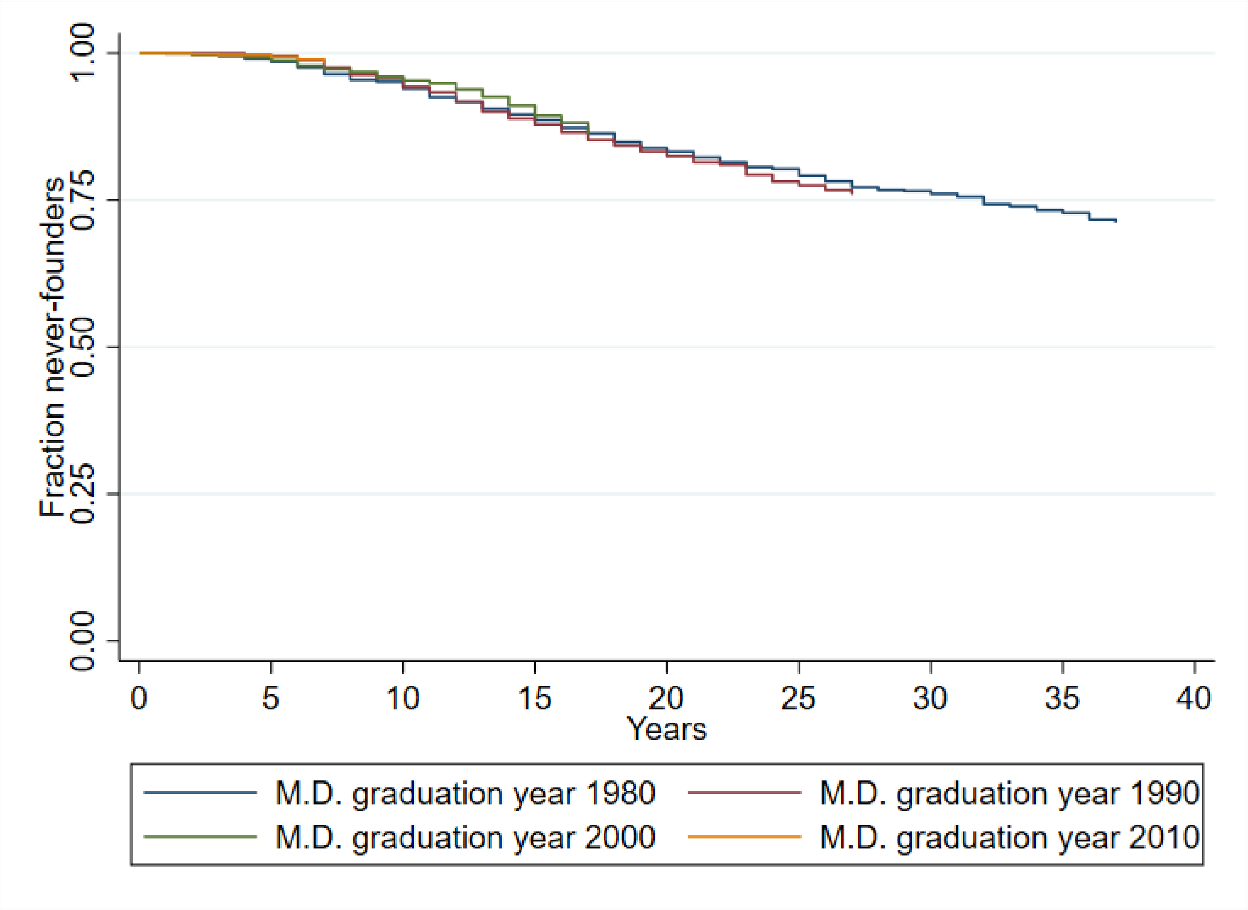

Note: Kaplan-Meier survival curves are shown where an event is having ever founded a company.

© 2021 Greenblatt. *JAMA Network Open*.

eFigure 6: Gender disparity by type of entrepreneurship

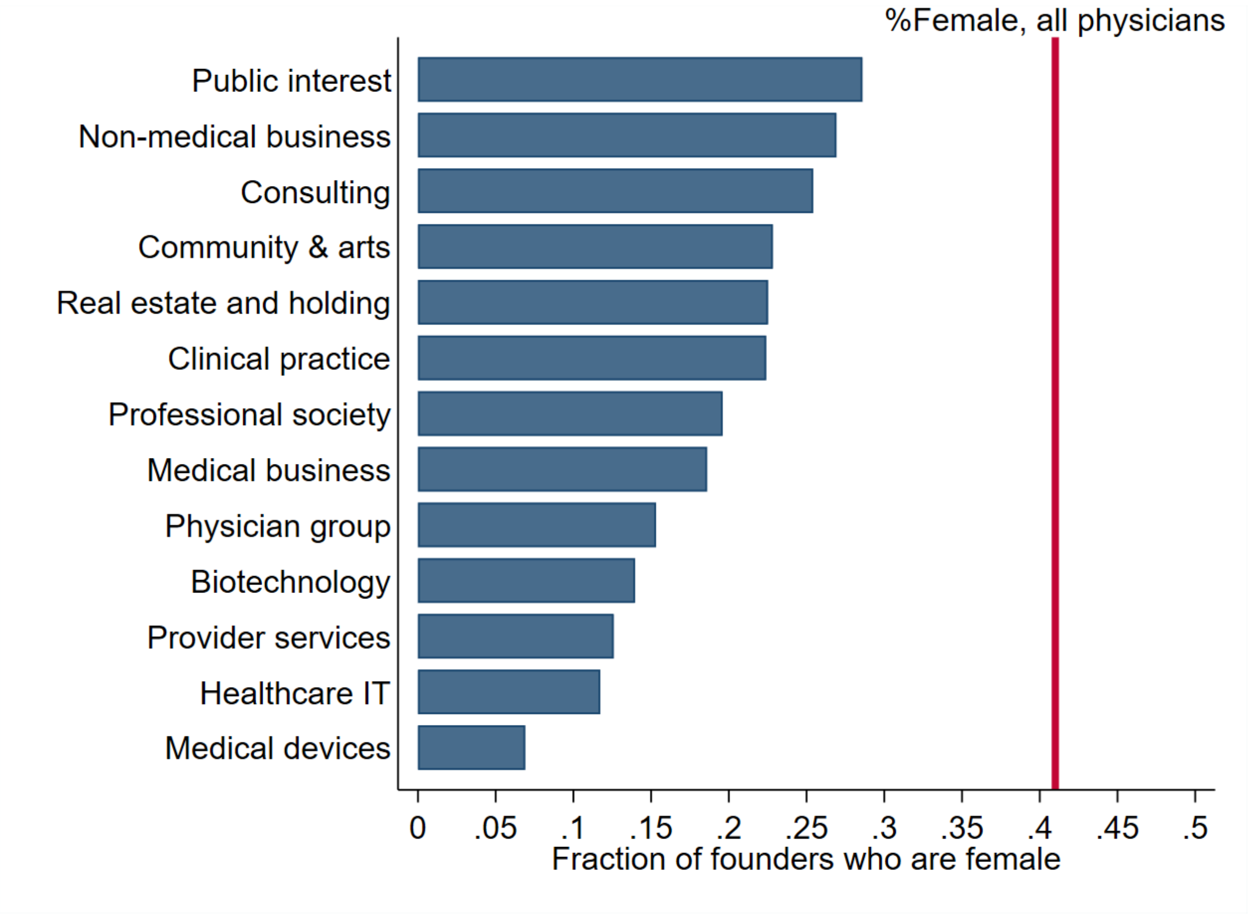

Note: Reports the fraction of physician founders of a given business type who are female. The vertical red line corresponds to the 41.0% of the overall Massachusetts physician population who are female.

© 2021 Greenblatt. *JAMA Network Open*.

eTable 1: Overview of business types

Type of startup	Description	Example
Biotechnology	Research and development of new diagnostic or therapeutic technologies	LQT Therapeutics, Inc.
Clinical practice	To provide direct patient care	Neurology Group of Worcester, P.D.
Community & arts	Organizations which support local community activities or affinity groups. Common examples include alumni societies, fine and performing arts, sports teams, religious organizations, and care of neighborhood resources	Mercury Orchestra, Inc.
Consulting	Self-described as consulting	Orchard Biomedical Consulting LLC
Healthcare IT	Development and marking of a software or digital product related to healthcare	Clinical Information Systems, Inc.
Medical business	A for-profit business which is closely related to medicine. Common examples include physician staffing companies, contract research organizations, retail medical products, and medical publishing	Commonwealth Clinical Studies, LLC
Medical devices	Development and marketing of physical devices for use in patient care, frequently with an associated patent	Ortho Scientia, Inc.
Non-medical business	A for-profit business which is far afield from medicine. Examples include restaurants, retail non-medical products, language schools, and sports analytics.	Romance Records/Wao Music, LLC
Other	Purpose is unclear from available documentation	Cari-Q, LLC
Physician group	Typically formed by a pre-existing hospital department and supports activities in clinical care, research and teaching	Beth Israel Deaconess Department of Neurology Foundation, Inc.
Professional society	Activities primarily targeted at healthcare providers to provide ongoing professional education and support	Society for Perioperative Assessment and Quality Improvement, Inc.
Provider services	Provides or supports clinical practice management or billing. Also includes accountable care organizations and companies contracting with health insurance payors	Physicians Billing Services, Inc.
Public interest	Primary purpose is closely related to the public good. This includes advocating for disadvantaged groups, public and global health including free medical care, and philanthropies raising money for charitable causes	The Boston Health Care for the Homeless Program, Inc.
Real estate & holding	To invest in or hold a real property or asset. Also includes ownership of ambulatory surgery or imaging facilities or capital equipment such as MRIs	299 Newport Avenue LLC

© 2021 Greenblatt. *JAMA Network Open*.

eTable 2: Physician characteristics

	Mean	Std. Dev.	Min.	Max.	Count
Physician characteristics					
Female	0.410	0.492	0	1	13,839
M.D. graduation year	1993.181	13.370	1946	2015	
Mass. licensure year	1999.942	13.395	1952	2017	
Time, M.D. to Mass. licensure (years)	6.759	6.275	0	52	
Medical school attended					
Massachusetts location	0.215	0.411	0	1	7,254
New England location	0.282	0.450	0	1	9,536
International medical graduate	0.238	0.426	0	1	8,029
NIH Funding ($2017)	252,273,081	373,101,368	0	1,480,734,825	
Log NIH Funding ($2017)	18.493	1.617	0	21.116	
Top 10 in U.S. NIH funding	0.162	0.369	0	1	5,483
Current address					
Massachusetts	0.807	0.394	0	1	27,265
New England	0.852	0.355	0	1	28,761
Specialty					
Anesthesiology	0.055	0.229	0	1	1,873
Dermatology	0.017	0.130	0	1	577
Emergency medicine	0.049	0.216	0	1	1,656
Family medicine	0.052	0.223	0	1	1,770
General surgery	0.045	0.208	0	1	1,536
Internal medicine	0.159	0.366	0	1	5,384
Medical subspecialties	0.162	0.368	0	1	5,462
Neurology	0.033	0.179	0	1	1,114
Neurosurgery	0.006	0.079	0	1	213
Obstetrics & gynecology	0.038	0.192	0	1	1,290
Ophthalmology	0.024	0.153	0	1	807
Orthopedics	0.028	0.165	0	1	950
Other	0.016	0.126	0	1	547
Otolaryngology	0.011	0.102	0	1	355
Pathology	0.029	0.167	0	1	973
Pediatrics	0.062	0.241	0	1	2,087
Pediatric subspecialties	0.038	0.192	0	1	1,298
Plastic surgery	0.008	0.089	0	1	270
Psychiatry	0.085	0.278	0	1	2,855
Radiation oncology	0.008	0.089	0	1	267
Radiology	0.064	0.244	0	1	2,154
Urology	0.010	0.099	0	1	332

Note: Descriptive statistics for the 33,770 physicians holding a Massachusetts medical license in 2017.

© 2021 Greenblatt. *JAMA Network Open*.

eTable 3: Physician characteristics by founding status

	Founders		Non-founders			
	**Mean**	**Std. Dev.**	**Mean.**	**Std. Dev.**	**Difference**	**P value**
Physician characteristics						
Female	0.244	0.430	0.449	0.497	0.205	<0.001
M.D. graduation year	1984.576	11.867	1995.230	12.883	10.653	<0.001
Mass. licensure year	1990.555	12.615	2002.177	12.581	11.622	<0.001
Time, M.D. to Mass. licensure (years)	5.978	5.221	6.945	6.487	0.966	<0.001
Medical school attended						
Massachusetts location	0.261	0.439	0.204	0.403	0.057	<0.001
New England location	0.328	0.469	0.272	0.445	0.056	<0.001
International medical graduate	0.239	0.426	0.238	0.426	0.001	0.85
NIH Funding ($2017)	259,195,545	392,743,667	250,627,110	368,267,531	8,568,435	0.15
Log NIH Funding ($2017)	18.531	1.443	18.484	1.656	0.048	0.06
Top 10 in U.S. NIH funding	0.163	0.369	0.162	0.369	0.001	0.93
Current address						
Massachusetts	0.907	0.290	0.784	0.412	0.123	<0.001
New England	0.930	0.254	0.833	0.373	0.097	<0.001
Specialty						
Anesthesiology	0.050	0.217	0.057	0.232	0.007	0.02
Dermatology	0.027	0.162	0.015	0.120	0.012	<0.001
Emergency medicine	0.044	0.206	0.050	0.218	0.006	0.05
Family medicine	0.056	0.230	0.052	0.221	0.004	0.14
General surgery	0.044	0.204	0.046	0.209	0.002	0.41
Internal medicine	0.147	0.354	0.162	0.369	0.015	0.003
Medical subspecialties	0.163	0.369	0.161	0.368	0.001	0.82
Neurology	0.025	0.155	0.035	0.184	0.010	<0.001
Neurosurgery	0.009	0.096	0.006	0.075	0.004	0.001
Obstetrics & gynecology	0.045	0.207	0.037	0.188	0.008	<0.001
Ophthalmology	0.046	0.210	0.019	0.135	0.027	<0.001
Orthopedics	0.046	0.210	0.024	0.153	0.022	<0.001
Other	0.025	0.156	0.014	0.118	0.011	<0.001
Otolaryngology	0.017	0.131	0.009	0.094	0.009	<0.001
Pathology	0.018	0.134	0.031	0.174	0.013	<0.001
Pediatrics	0.055	0.228	0.063	0.244	0.008	<0.001
Pediatric subspecialties	0.018	0.133	0.043	0.204	0.025	<0.001
Plastic surgery	0.016	0.127	0.006	0.077	0.010	<0.001
Psychiatry	0.083	0.276	0.085	0.279	0.002	0.65
Radiation oncology	0.006	0.074	0.008	0.092	0.003	0.02
Radiology	0.043	0.202	0.069	0.253	0.026	<0.001
Urology	0.017	0.129	0.008	0.090	0.009	<0.001

Note: Descriptive statistics, by founding status, for the 33,770 physicians holding a Massachusetts medical license in 2017.

© 2021 Greenblatt. *JAMA Network Open*.

eTable 4: Entrepreneurship outcomes: Logistic regression

	(1) Any	(2) Clinical practice	(3) Public interest	(4) Biotech	(5) Medical devices	(6) Healthcare IT	(7) Medical business	(8) Non-medical business
Female	0.529***	0.506***	0.790**	0.319***	0.162***	0.207***	0.447***	0.732***
	(0.018)	(0.022)	(0.077)	(0.083)	(0.055)	(0.049)	(0.046)	(0.082)
International medical graduate	1.090**	1.127***	1.176	1.272	1.030	1.056	1.218**	1.778***
	(0.042)	(0.052)	(0.123)	(0.344)	(0.244)	(0.210)	(0.122)	(0.199)
Massachusetts medical school	1.421***	1.329***	1.484***	1.654***	1.079	1.487**	1.386***	1.397***
	(0.053)	(0.060)	(0.142)	(0.319)	(0.224)	(0.251)	(0.130)	(0.166)
Top 10 research school	0.950	0.687***	1.519***	4.326***	1.696***	1.457**	1.020	1.056
	(0.041)	(0.038)	(0.155)	(0.845)	(0.344)	(0.275)	(0.111)	(0.145)
Anesthesiology	0.856**	0.846*	0.331***	0.747	2.507**	0.744	0.487***	1.132
	(0.065)	(0.079)	(0.098)	(0.425)	(1.103)	(0.286)	(0.113)	(0.234)
Dermatology	2.385***	2.840***	0.719	1.659	4.732***	1.341	2.088***	0.821
	(0.267)	(0.367)	(0.251)	(1.082)	(2.652)	(0.725)	(0.480)	(0.326)
Emergency medicine	0.972	0.832*	0.960	1.170	1.130	1.372	1.556***	1.354
	(0.076)	(0.083)	(0.206)	(0.611)	(0.678)	(0.452)	(0.259)	(0.294)
General surgery	0.820**	0.691***	0.695	1.004	3.440***	0.547	0.570**	0.843
	(0.065)	(0.069)	(0.156)	(0.502)	(1.448)	(0.250)	(0.128)	(0.202)
Medical subspecialties	0.825***	0.744***	0.922	2.657***	1.434	1.033	0.675***	0.593***
	(0.044)	(0.049)	(0.126)	(0.812)	(0.551)	(0.258)	(0.091)	(0.101)
Neurology	0.683***	0.592***	0.988	2.141	0.407	1.545	0.687	0.576*
	(0.067)	(0.075)	(0.227)	(0.998)	(0.427)	(0.574)	(0.174)	(0.188)
Neurosurgery	1.365*	1.010	1.204	2.697	6.971***	1.085	0.607	1.756
	(0.227)	(0.211)	(0.489)	(1.735)	(3.919)	(0.795)	(0.314)	(0.725)
Obstetrics & gynecology	1.285***	1.694***	0.762	1.980	3.156**	1.172	0.807	0.876
	(0.103)	(0.155)	(0.182)	(0.980)	(1.581)	(0.498)	(0.184)	(0.231)
Ophthalmology	2.416***	2.515***	0.942	2.692**	6.347***	1.103	2.109***	1.339
	(0.213)	(0.251)	(0.237)	(1.260)	(2.764)	(0.504)	(0.386)	(0.351)
Pathology	0.518***	0.413***	0.574*	1.698	2.361	0.176*	0.475**	0.386**
	(0.057)	(0.062)	(0.170)	(0.899)	(1.292)	(0.180)	(0.146)	(0.153)
Pediatrics	1.027	1.204**	0.892	0.218	0.329	1.284	0.327***	0.565**
	(0.075)	(0.106)	(0.175)	(0.227)	(0.347)	(0.462)	(0.094)	(0.150)
Pediatric subspecialties	0.439***	0.315***	0.903	1.085	1.301	0.469	0.459***	0.346***
	(0.047)	(0.049)	(0.208)	(0.625)	(0.856)	(0.286)	(0.135)	(0.137)
Psychiatry	0.747***	0.728***	0.925	0.489	0.288	0.804	0.750*	0.723*
	(0.049)	(0.059)	(0.145)	(0.266)	(0.225)	(0.267)	(0.122)	(0.142)

© 2021 Greenblatt. *JAMA Network Open*.

Radiology	0.597***	0.598***	0.308***	0.475	0.595	0.651	0.449***	0.769
	(0.046)	(0.058)	(0.089)	(0.303)	(0.397)	(0.251)	(0.102)	(0.174)
Surgical subspecialties	1.778***	1.840***	0.940	0.406	5.959***	0.865	1.052	1.252
	(0.117)	(0.141)	(0.171)	(0.259)	(2.225)	(0.292)	(0.173)	(0.242)
Constant	0.699***	0.433***	0.057***	0.005***	0.004***	0.006***	0.040***	0.031***
	(0.092)	(0.064)	(0.016)	(0.003)	(0.003)	(0.005)	(0.014)	(0.011)
N	33,770	33,770	33,770	33,770	33,770	33,770	33,770	33,770
Pseudo R^2	0.145	0.151	0.073	0.148	0.139	0.062	0.092	0.062
Log likelihood	-14,137	-10,194	-2,961	-754	-805	-1,119	-3,083	-2,361

Note: All estimates stem from logistic regression with results reported as odds ratios. The dependent variable is a binary variable for having ever founded a firm of the given business type. Internal medicine is the omitted category, so specialty coefficients should be interpreted relative to internal medicine. All models also include an indicator variable for other specialties as well as a set of 11 indicator variables for five-year periods of medical school graduation year. Robust errors in parentheses (* $p < 0.10$, ** $p < 0.05$, *** $p < 0.01$).

© 2021 Greenblatt. *JAMA Network Open*.

eTable 5: Alternative logistic model specifications

**Dependent variable: Ever founder (any type)**	**(1)**	**(2)**	**(3)**	**(4)**	**(5)**	**(6)**
Female	0.396***	0.393***	0.384***	0.529***	0.514***	0.511***
	(0.012)	(0.012)	(0.013)	(0.019)	(0.021)	(0.021)
International medical graduate	1.005	1.119***	1.215***	1.081**		
	(0.033)	(0.040)	(0.045)	(0.041)		
Mass. medical school		1.466***	1.475***	1.423***	1.426***	
		(0.051)	(0.052)	(0.053)	(0.055)	
Top 10 research school		0.962	0.964	0.952		
		(0.038)	(0.039)	(0.041)		
Log NIH funding					1.002	
					(0.010)	
Constant	0.326***	0.294***	0.267***	0.448**	0.389*	0.501
	(0.006)	(0.007)	(0.011)	(0.178)	(0.188)	(0.231)
Specialty controls	No	No	Yes	Yes	Yes	Yes
Graduation year controls	No	No	No	Yes	Yes	Yes
U.S. medical school controls	No	No	No	No	No	Yes
N	33,770	33,770	33,770	33,770	25,741	25,741
Pseudo R^2	0.029	0.033	0.053	0.149	0.162	0.168
Log likelihood	-16,051	-15,991	-15,662	-14,073	-10,549	-10,473

Note: All estimates stem from logistic regression with results reported as odds ratios. Specifications 5 and 6 limit the sample to only those physicians who graduated from a U.S. medical school. Estimates also include a full set of indicator variables for clinical specialties, medical school graduation years, and U.S. medical schools as noted. Robust errors in parentheses (* $p < 0.10$, ** $p < 0.05$, *** $p < 0.01$).

© 2021 Greenblatt. *JAMA Network Open*.
